# High-Density Glass Scintillators for Proton Radiography—Relative Luminosity, Proton Response, and Spatial Resolution

**DOI:** 10.3390/s24072137

**Published:** 2024-03-27

**Authors:** Ethan Stolen, Ryan Fullarton, Rain Hein, Robin L. Conner, Luiz G. Jacobsohn, Charles-Antoine Collins-Fekete, Sam Beddar, Ugur Akgun, Daniel Robertson

**Affiliations:** 1 Department of Radiation Oncology, Mayo Clinic, Phoenix, AZ 85054, USA; ethanstolen@uchicago.edu; 2 Department of Medical Physics and Biomedical Engineering, University College London, London WC1E 6BT, UK; ryan.fullarton.20@ucl.ac.uk (R.F.); c.fekete@ucl.ac.uk (C.-A.C.-F.); 3 Department of Physics, Coe College, Cedar Rapids, IA 52402, USA; cahein22@coe.edu (R.H.); uakgun@coe.edu (U.A.); 4 Department of Materials Science and Engineering, Clemson University, Clemson, SC 29634, USA; rlconne@g.clemson.edu (R.L.C.); luiz@g.clemson.edu (L.G.J.); 5 Graduate School of Biomedical Sciences, University of Texas MD Anderson Cancer Center, Houston, TX 77030, USA; abeddar@mdanderson.org

**Keywords:** proton radiography, proton therapy, glass scintillator, imaging, scintillator characterization

## Abstract

Proton radiography is a promising development in proton therapy, and researchers are currently exploring optimal detector materials to construct proton radiography detector arrays. High-density glass scintillators may improve integrating-mode proton radiography detectors by increasing spatial resolution and decreasing detector thickness. We evaluated several new scintillators, activated with europium or terbium, with proton response measurements and Monte Carlo simulations, characterizing relative luminosity, ionization quenching, and proton radiograph spatial resolution. We applied a correction based on Birks’s analytical model for ionization quenching. The data demonstrate increased relative luminosity with increased activation element concentration, and higher relative luminosity for samples activated with europium. An increased glass density enables more compact detector geometries and higher spatial resolution. These findings suggest that a tungsten and gadolinium oxide-based glass activated with 4% europium is an ideal scintillator for testing in a full-size proton radiography detector.

## 1. Introduction

### 1.1. Developments in Proton Radiography

The clinical implementation of proton therapy has grown tremendously since Wilson [1] first proposed using high-energy protons therapeutically in 1946, perhaps due to its capacity for sparing normal tissue, such as in treatments for head and neck cancer or pediatric cancer [2,3,4,5,6,7,8,9]. Protons deposit the highest dose near the end of their range, a distribution described by the Bragg peak, allowing delivery of the maximum dose to the target while minimizing the dose to surrounding tissues [10]. However, widespread clinical implementation is hindered by several factors, including the high equipment cost and errors in proton range calculations [6,11,12]. Proton therapy treatment planning still relies on X-ray CT data to obtain the electron density inside the patient, which is converted into proton stopping power, usually by a stoichiometric calibration [13]. This imperfect conversion introduces uncertainty in the proton range of up to 5% [14]. The development of proton radiography could reduce this source of error during treatment planning by eliminating the need to convert from electron density to proton stopping power. There are some disadvantages in imaging with protons compared to X-rays, such as increased multiple Coulomb scattering of protons leading to reduced image resolution [15]. Despite the challenges, the need for an exact measurement of the proton stopping power in patients and the prospect of imaging with the radiation source used during treatment continue to motivate the development of proton radiography.

The most extensively studied practical system for proton radiography includes using particle trackers to measure a single proton’s position before and after entering the patient, as well as the residual proton energy after transiting the patient. Several proof-of-concept studies have investigated proton radiography using single-particle tracking systems, demonstrating the superior image quality resulting from the ability to model the effects of multiple Coulomb scattering, including the first proton radiograph of an animal [16,17,18,19]. However, single-particle tracking systems require high-speed instrumentation, which can be costly and impractical in the clinic. Additionally, these systems require a lower proton beam fluence, on the order of 10^6^ protons per second or lower, due to the limitations of the detector electronics, compared to the typical clinical proton fluence of 10^9^ protons per second [20,21]. Modifying the clinical proton beam to accommodate this lower proton fluence as proposed by some studies is another impractical and costly barrier to implementing single-particle tracking radiography [22,23].

An alternative method of proton radiography creates images by integrating the energies of all protons that traverse the patient. Integrating-mode detectors have limited ability to account for proton scattering, causing them to lack the exquisite spatial resolution of single-particle-tracking detectors. However, they can accommodate clinically used proton fluences, making them much easier to integrate into clinical proton therapy systems [24]. They are also much simpler in terms of instrumentation. Most studies of proton-integrating systems have used flat imaging panels, which require modulations in the range or energy of the beam to create the radiograph [25,26,27,28,29,30], resulting in a higher dose to the patient and longer image acquisition times [24]. To address this issue, Tanaka et al. [31] demonstrated a proton radiography system that uses a scintillator block to measure the energies of all protons. A complementary metal–oxide–semiconductor (CMOS) camera measures the visible light the scintillator emits, which relates to the proton dose.

Our research group has developed large-volume scintillator detectors for dosimetry and characterization of clinical proton beams using liquid scintillators [32,33,34,35]. Researchers have also applied these detectors to produce integrating-mode proton radiographs using the commercial liquid scintillator OptiPhase HiSafe 3 (PerkinElmer, Waltham, MA, USA) [36] and plastic scintillator EJ-260 (Eljen Technology, Sweetwater, TX, USA) [24]. These investigations have demonstrated the usefulness of a monolithic scintillator-based design and established the need to further investigate the optimal scintillator material and system design. A major limitation of scintillator-based detectors is the large dimensions of the scintillator block in the depth direction, which is required to stop protons of the highest clinical energies, which can be as high as 250 MeV and have ranges of up to 38 cm in water. A novel, high-density scintillating glass will reduce the required thickness of the scintillator, making the system easier to install on existing proton therapy gantries. Additionally, stopping the protons more quickly may decrease the impact of proton scattering in the detector, potentially leading to an increase in spatial resolution.

### 1.2. High-Density Scintillating Glass

Compared with other available scintillating materials, high-density activated glass scintillators have several advantages. Glass scintillators typically have densities between those of low-density organic scintillators and higher-density inorganic crystal scintillators [37]. The first examples of scintillating glasses, containing SiO_2_, MgO, Al_2_O_3_, Ce_2_O_3_, and Li_2_O, had densities around 4 g/cm^3^ [38,39]; however, subsequently developed scintillating glasses containing Gd_2_O_3_ and Lu_2_O_3_ have achieved densities of more than 5 g/cm^3^ [40,41]. A scintillating material with a higher density can stop protons with less thickness than an organic scintillator, because there exist a greater number of atoms per unit thickness which absorb the energy of the proton [42]. Thus, a proton radiography detector with a glass scintillator will require significantly less thickness than the organic scintillators described in the previous section.

Conversely, although glass scintillators have lower densities compared to inorganic crystal scintillators, glass scintillators also have several advantages in proton radiography applications, such as easier manufacturing in various shapes and sizes. Most proton radiography systems with inorganic scintillator detectors would need to be made up of many single crystals, since most inorganic crystals cannot be grown larger than a few centimeters [37]. The result is reduced optical efficiency as light passes between imperfectly joined crystals. Glass scintillators are easy to manufacture in many different shapes and sizes, with considerably lower cost of production than inorganic crystals [42,43,44]. Some glass scintillators have also exhibited relatively short decay times [37], an especially important consideration in single-particle tracking systems, which require extremely fast electronics and scintillator response. A shorter decay time may also be useful in the proton-integrating approach, potentially decreasing image acquisition times when multiple images are needed, such as for proton computed tomography (pCT) [15].

We have previously developed several high-density scintillating glasses, specifically for proton radiography applications, activated with cerium, europium, and terbium, with densities ranging between 4.19 g/cm^3^ and 5.84 g/cm^3^ [45]. We determined that glasses activated with europium possessed favorable optical properties, such as transparency, and higher relative luminosity, making them especially promising for use in a proton radiography detector. We have also described the manufacturing process for these glass scintillators, which is significantly more cost effective than the manufacturing process for inorganic crystal scintillators.

We continued this work by testing a proof-of-concept pCT detector using Monte Carlo simulations [46]. The prototype detector used the scintillating glass activated with europium, which had the most promising optical properties from the pCT image reconstruction study. We successfully reconstructed simulated pCT images, demonstrating the feasibility of their pCT detector system based on a single-particle-tracking detector. However, we have yet to test the proton response of these scintillating glasses in a physical experiment. The goal of this work is to measure the physical response to proton irradiation of several of these scintillating glasses, including relative luminosity, a quantification of the ionization quenching in each sample, and the impact of the material on proton radiograph spatial resolution, with the goal of confirming their feasibility and selecting the optimal glass for constructing a scintillator-based detector for proton radiography.

## 2. Materials and Methods

We selected three different base glasses, two of which consisted of various percentages of tungsten and gadolinium oxides to achieve higher densities. For comparison, a base glass with a lower density, containing lead oxide, was selected. Adding different ratios of europium (III) oxide or terbium (III) oxide to the base glasses produced six different activated scintillator samples, summarized in Table 3. Our previous studies showed that the 4% Eu concentration had greater relative luminosity than 5%, 6%, or 7% Eu concentration in similar high-density glasses [45]. The same was true for Tb. Based on these results, we limited the concentrations of Eu and Tb to a maximum of 4% in this study.

Some image reconstruction methods for integrating-mode proton radiography require an accurate representation of the depth–dose curve, which is obscured by quenching effects in the scintillator detector [47]. Therefore, we determined the ionization quenching of each glass scintillator by comparing depth–light curves from each scintillator sample to the depth–dose and depth–linear energy transfer (LET) curves generated by Monte Carlo simulations of the interaction of a proton beam with the scintillator samples.

### 2.1. Physical Measurements

#### 2.1.1. Ionization Quenching Measurement

We manufactured solid glass scintillator samples by pouring molten glass at 500 °C into a brass mold to form a set of cylindrical samples with uniform radius, height, and volume. We further polished the glass samples to improve their uniformity and transparency. The average radius and height of the scintillator samples were 14.4 mm and 3.2 mm, respectively.

**Figure 1:**
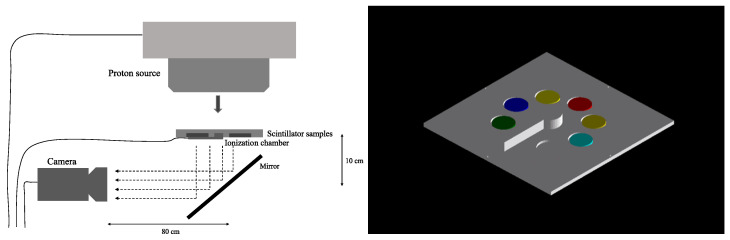
Experimental setup diagram on the proton therapy gantry at Mayo Clinic Arizona (**left**), and the experimental plate and scintillators as modeled in TOPAS MC (**right**).

To generate the depth–light curves for each of the six scintillator samples, we designed a detector plate that would ensure that the scintillating glass samples would be in the plane perpendicular to the beam and at the same water-equivalent depth, along with an Advanced Markus ionization chamber (PTW, Freiburg, Germany) for measuring the depth–dose curve (see Figure 1). The experimental plate was manufactured with the Ultimaker S5 extrusion 3D printer (Utrecht, The Netherlands), which has a layer accuracy of 0.2 mm. The detector plate was specifically designed to be mounted on an XRV-2000 Falcon Beam Profiler (Logos Vision Systems, Scotts Valley, CA, USA), which is equipped with a mirror that directs scintillation light from the plate to a built-in CMOS camera. To ensure the accuracy of our measurements by avoiding the contamination of light from other sources, including PMMA scintillation, the scintillator holder plate and camera were enclosed in a light-tight enclosure within the XRV-2000 Falcon Beam Profiler.

**Figure 2:**
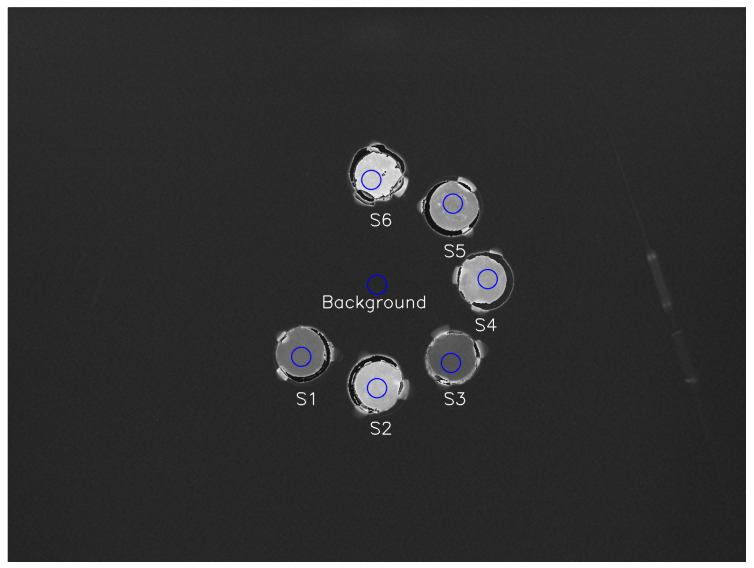
Image of the scintillator samples during irradiation, taken by the CMOS camera at a water-equivalent depth of 132 mm, the position of the Bragg peak. The ROIs used to measure average pixel intensity are outlined in blue.

We measured the depth–light response to a beam of 144.8 MeV protons, which is in the middle of the energies produced by the accelerator at Mayo Clinic. The Hitachi ProBeat proton accelerator (Tokyo, Japan) delivered a uniform fluence of 144.8 MeV protons to a 15 cm square field centered on the experimental plate. By placing different thicknesses of polymethyl methacrylate (PMMA) in front of the detector and measuring the resulting relative luminosity with the CMOS camera, the depth–light curve was measured for each scintillator. We extracted the relative luminosity from equally sized regions of interest (ROIs) in the CMOS camera images corresponding to the position of each scintillator, as well as one corresponding to a measurement of mean background pixel intensity, which was subtracted from the mean pixel intensity of each scintillator. The positions of the ROIs were manually adjusted to avoid areas of uneven relative luminosity in the samples, as seen in Figure 2. The depth–light curve was normalized to the relative luminosity at the entrance depth.

#### 2.1.2. Relative Luminosity Measurement

We measured relative luminosity by crushing the scintillator samples to powder and comparing their luminosity to that of bismuth germanium oxide (BGO, 99.9995%, metals basis, Alfa Aesar Puratronic, Thermo Fisher Scientific, Waltham, MA, USA) scintillator powder, which is well characterized in the literature [48,49]. The relative luminosity measurements were made using a custom-designed configuration of the Freiberg Instruments Lexsyg spectrofluorometer (Freiberg, Germany) equipped with a Varian Medical Systems VF-50J X-ray tube (Palo Alto, CA, USA) with a tungsten target, coupled with a Crystal Photonics CXD-S10 photodiode (Berlin, Germany) for continuous radiation intensity monitoring. The light emitted by the sample was collected by an Andor Technology (Belfast, UK) system comprised of a SR-OPT-8024 optical fiber, Shamrock 163 spectrograph, and a cooled (−80 °C) DU920P-BU Newton CCD camera (spectral resolution of ~0.5 nm/pixel). Relative luminosity was measured under continuous X-ray irradiation (W lines and bremsstrahlung radiation; 40 kV, 1 mA) with an integration time of 5 s. The powdered glass samples and BGO powder filled ~8 mm diameter, 0.5 mm deep cups, allowing for comparison of equal volumes and the determination of relative luminosity. The relative luminosity results correspond to the ratio of the spectra integral from 300 to 750 nm using the BGO spectrum integral as reference.

### 2.2. Monte Carlo Simulations

#### 2.2.1. Ionization Quenching

Ionization quenching describes the nonlinear response of scintillators to the energy of particles as a function of linear energy transfer (LET) [50]. Birks’s quenching theory [51,52], described by Equation (1), relates luminosity to proton LET, where *S* is the scintillator luminosity, *A* is the scintillation efficiency, $\frac{dE}{dx}$ is the proton LET, and kB is Birks’s constant.

(1) $$\frac{dS}{dx}=\frac{A\frac{dE}{dx}}{1+kB\frac{dE}{dx}}$$

To fully quantify the quenching effect in each scintillator sample, we compared the measured depth–light curves to depth–dose and depth–LET curves generated through Monte Carlo simulations. The chemical compositions of the six scintillating glasses, as well as PMMA and polylactic acid (PLA) that made up the experimental sample plate, were modeled in the TOPAS MC wrapping of the Geant4 simulation toolkit [53]. The parameter details of the Monte Carlo simulations are included in Table 1. The simulated experimental plate and scintillator samples matched the exact dimensions and material composition of the physical experiment (Figure 1, right). The proton LET calculations allowed for the calculation of the Birks constant in each sample.

#### 2.2.2. Beam Range and Spatial Resolution

We also modeled the glass scintillators directly in the Monte Carlo radiation transport code Geant4 [55] to measure the beam range and spatial resolution of integrating proton radiographs acquired using the scintillators. The commercially available plastic scintillator EJ-260 (Eljen Technologies, Sweetwater, TX, USA) was also modeled in Geant4 for comparison to currently used scintillators. A simulation of a single pencil beam in a block of each material was performed in order to evaluate the range and lateral profile of the proton beam in each material. The details of the Monte Carlo simulations are given in Table 2. The materials for samples 1 and 5 were modeled according to the compositions listed in Table 3, and the proton beam was given an energy of 200 MeV, a sigma of 2 mm, and 5 mrad of beam divergence.

To evaluate the impact of the materials on the spatial resolution, we created a digital phantom, including a 4 cm thick Al slab placed in a 10 cm deep water tank, with the slab’s surface 4 cm behind the surface of the water tank. The slab was rotated to an angle of 2.5° to provide the slanted-edge data required for analysis of the modulation transfer function according to the ISO 12233 standard [60] for measuring spatial resolution in digital cameras. A block of scintillator was placed behind the water tank and Al slab, and the radiation dose within the scintillator was summed in the beam direction. This dose summation is a surrogate for the light distribution in the scintillator, forming an integrating proton radiograph. The simulation was repeated for the glass scintillator samples 1 and 5, as well as a block of EJ-260.

## 3. Results

### 3.1. Relative Luminosity

Europium-activated glasses demonstrated higher relative luminosity compared to terbium-activated glasses, yielding a relative luminosity of 5–8% of BGO for europium glasses and 2–4% for terbium glasses. Samples S1 and S2, which have the same glass composition and activation element concentration, demonstrate the highest relative luminosity of samples with activation by Eu_2_O_3_. In samples containing europium (Eu_2_O_3_), the relative luminosity increased with the concentration of the activation element. Sample S6, containing 4% Eu_2_O_3_, demonstrated the highest relative luminosity (8% of BGO) among all glass samples. The terbium glasses appear to demonstrate concentration quenching, with sample S5 (3% terbium) producing a higher relative luminosity than sample S1 (4% terbium). Figure 2 shows the image of the scintillator samples at a water-equivalent depth of 132 mm, which is the observed position of the Bragg peak.

**Figure 3:**
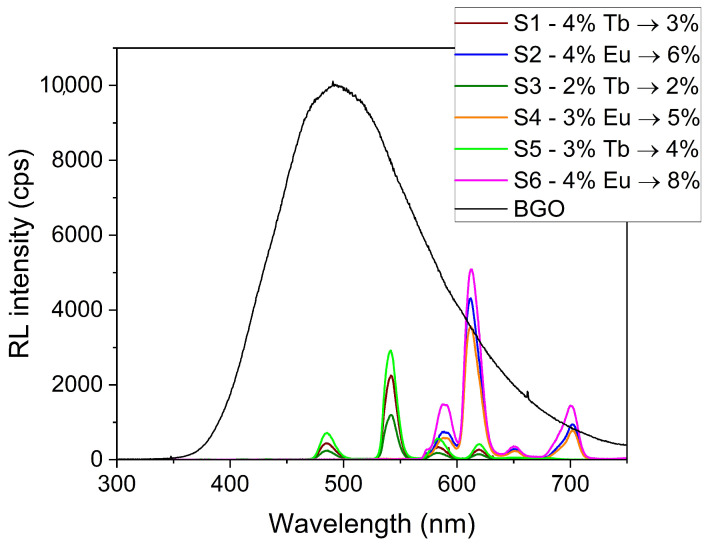
The emission spectra of the scintillator samples and BGO powder.

The spectral response of the terbium-activated glasses was greater at shorter wavelengths (Figure 3), giving them an advantage when paired with photomultiplier tubes, which are more sensitive at shorter wavelengths. The spectral response of the europium-activated glasses was stronger at longer wavelengths, making these glasses better suited to camera-based measurement (e.g., with CCD or CMOS cameras, which typically exhibit peak quantum efficiency in the 600–700 nm region).

### 3.2. Ionization Quenching

The depth–light response of the scintillator samples clearly displays ionization quenching, with the ratio of the peak to plateau of the lumninosity being 33–37% less than that of the ionization chamber [61]. The data also demonstrate the impact of the type and concentration of the activation element on the Birks correction factor of the glass scintillators, as shown in Table 3 and Figure 3.

**Figure 4:**
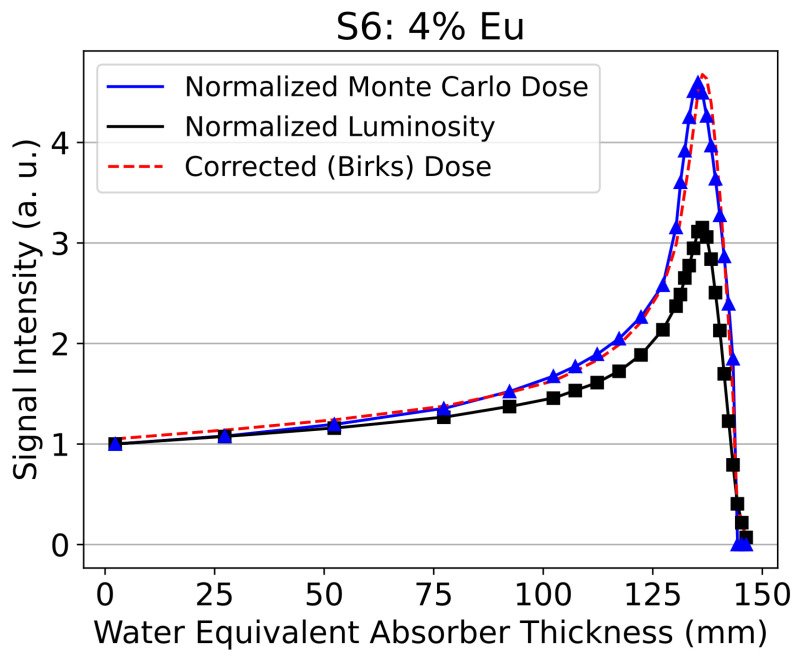
Depth–light curve for sample S6, which displayed the highest relative luminosity. The normalized depth–light curve (black) and corrected depth–dose curve (red) is compared to the Monte Carlo calculated depth–dose curve (blue).

Figure 4 shows the extent of ionization quenching in the relative luminosity (black) of sample 6 compared to the calculated dose (blue). In terms of Birks correction factors, samples with Tb_2_O_3_ had higher factors, indicating that ionization quenching might be more significant for scintillators activated with Tb_2_O_3_. Figure 4 demonstrates the success of Birks’s equation for correcting ionization quenching for the sample with the highest relative luminosity, S6, which is shown in the dashed red line and very closely matches the calculated dose in blue.

The steep rise in the depth–LET curve can substantially impact the quenching correction results in the presence of discrepancies between measured and modeled depth–light and depth–dose curves. To minimize any setup error’s impact, we measured the goodness of fit (R^2^) for the Birks corrected depth–dose curve for different shifts in the Monte Carlo data from the experimental data and averaged across all samples [62,63]. We computed the average R^2^ for shifts ranging from −2 mm to 2 mm in 0.1 mm intervals. The average R^2^ value was highest for a shift of 0 mm, which indicates that the experimental data and Monte Carlo data were well aligned.

### 3.3. Beam Range and Spatial Resolution

**Figure 5:**
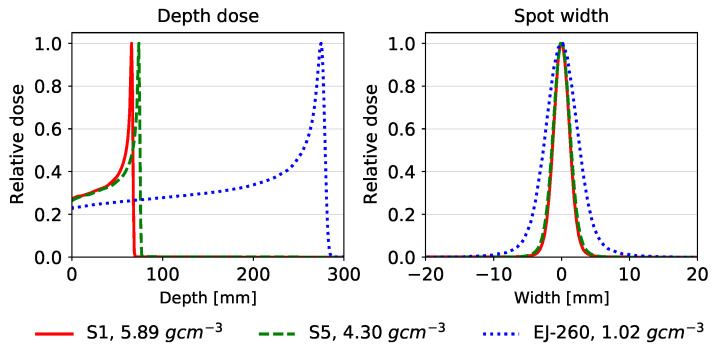
Depth–dose curves of a 200 MeV proton beam in Sample S1 (tungsten and gadolinium oxide glass), sample S5 (lead borate glass), and EJ-260 (organic plastic scintillator) (**left**). Lateral profiles at the Bragg peak of a 200 MeV proton beam for the three samples (**right**).

Our Monte Carlo calculations of proton pencil beams in blocks of different scintillator materials showed that the lead borate and tungsten/gadolinium oxide glasses decreased the proton beam range from 276 mm in EJ-260 to 75 mm in lead borate glass and 68 mm in the tungsten and gadolinium oxide glasses, as shown in Figure 5. Due to the rapid stopping in the glass scintillators, the proton beams exhibited less lateral scatter, leading to a decrease in the full width at half maximum (FWHM) at the Bragg peak from 16 mm for EJ-260 to 8 mm for the glass scintillator materials.

**Figure 6:**
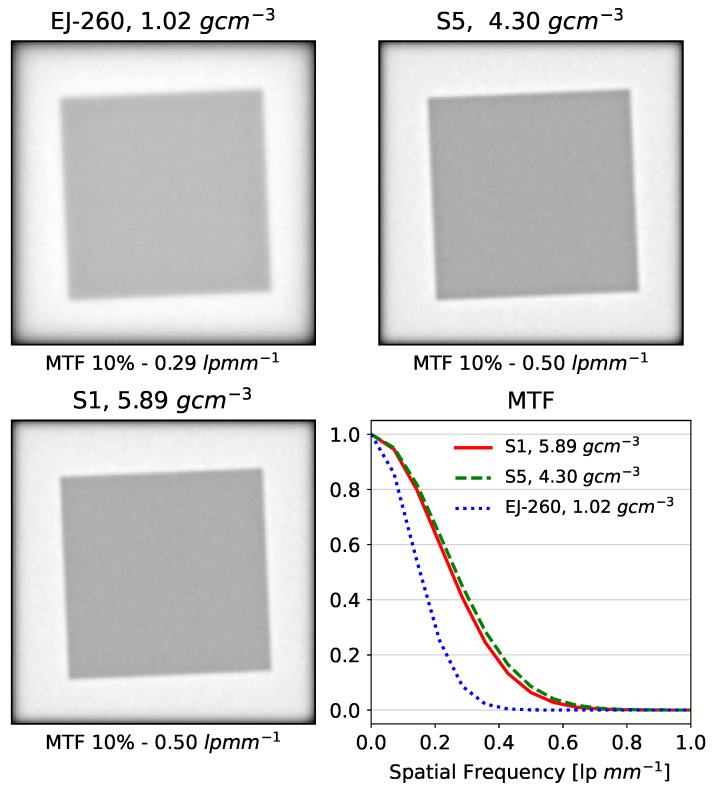
A 200 MeV proton field with dimensions 15 × 15 cm^2^ transported through a 4 cm thickness Al slab inside a 10 cm thickness water tank, with the residual proton energy being absorbed in a block of EJ-260 organic plastic scintillator (**upper left**), lead oxide glass scintillator (**upper right**), and tungsten and gadolinium oxide glass scintillator (**lower left**). The resulting modulation transfer function curves are shown (**lower right**).

The decreased scatter led, as expected, to increased spatial resolution, as measured by the simulation of beam transport through a tilted Al block in a water phantom (Figure 6). The EJ-260 block yielded a 10% modulation transfer function (MTF_10%_) of 0.29 line pairs per mm (lp/mm), while the lead oxide and tungsten–gadolinium oxide glass scintillators both yielded an MTF_10%_ of 0.50 lp/mm (Figure 6).

## 4. Discussion

The results obtained from this study have provided valuable insights into the scintillation properties of high-density glass scintillators. By analyzing the effects of different activation elements and their concentrations, the factors that influence the scintillation efficiency, relative luminosity, and ionization quenching of these materials are better understood. Specifically, this study has demonstrated the promise of europium as an activation element in glass scintillators for proton radiography.

Additionally, the observed differences in the Birks correction factors across the samples indicate that ionization quenching varies with the bulk material and activation elements of the glass scintillators. This finding highlights the importance of carefully considering the composition of scintillating glasses when designing proton radiography detectors to ensure optimal performance. The study also demonstrated the successful correction of the measured dose by Birks’s equation in glass scintillators.

The tested scintillating glass materials enable the stopping of clinical proton beams in 20% or 25% of the thickness required by conventional organic scintillators (for tungsten/gadolinium oxide and lead borate glasses, respectively), allowing construction of a more compact imaging device. The decrease in scattering caused by the shorter proton range also leads to enhanced spatial resolution, by a factor of 1.7, for both high-density glass materials relative to organic plastic scintillators.

This study has contributed to the ongoing efforts to develop more compact, cost-effective, and efficient proton radiography detectors. By identifying the trade-offs between density, scintillation efficiency, and ionization quenching, researchers can now make more informed decisions when selecting scintillator materials for full-size detectors. Nevertheless, it is important to recognize that further research is necessary to fully optimize scintillating glass materials for proton radiography applications. Future studies should focus on refining the synthesis methods, exploring alternative activation elements, and investigating other factors that may influence the scintillation properties and ionization quenching of these materials. This might include experimenting with other glass materials to produce glass scintillators with even greater density or higher relative luminosity. Additionally, studies investigating the response of these materials to different proton energies and beam configurations could provide further insights into their potential applications in diverse proton imaging modalities.

An exploration of the long-term stability and resistance to radiation damage, or radiation hardness, of these scintillating glasses, would also be useful, as these factors are crucial for ensuring the reliability and longevity of detectors in clinical and research settings. Research has found that Eu^3+^-activated glass scintillators have greater radiation hardness compared to Ce^3+^-activated glass scintillators [64,65]. The emission of Eu^3+^ is due to shielded 4f–4f transitions that are only sensitive to defects in their close vicinity [42], while Ce^3+^ emission is due to unshielded 5d–4f, which is more sensitive to radiation damage [66]. Since Tb^3+^ also exhibits emission due to 4f–4f transitions, it is likely that our glasses will posses moderate radiation hardness. However, further experiments would serve to confirm the radiation hardness of our particular glass scintillators. Such radiation stability information will be crucial in deciding which glass scintillators to install in a clinically used proton radiography system.

## 5. Conclusions

Our study has successfully demonstrated the promise of scintillating glasses as a viable alternative to organic scintillator materials for proton radiography detectors. We propose that a tungsten and gadolinium oxide-based glass activated with 4% europium combines the higher relative luminosity associated with europium-activated samples and the increased spatial resolution and small detector depth of higher-density glass. By building on the findings presented here, continued research in this area will lead to the development of advanced detectors that can meet the growing demands of the proton therapy field and contribute to the improvement in patient outcomes in cancer treatment.

## Figures and Tables

**Table 1:** TOPAS simulation parameters.

Parameter	Description	References
Code release date	TOPAS Version 3.8.1	[53]
Validation	Validated for proton transport applications	[54]
CPU description	High-performance cluster, 100 h computation time on 32 CPUs	
Source description	10 cm square field of 144.8 MeV monoenergetic protons	
Cross-sections	TOPAS default parameters	[53]
Transport parameters	TOPAS default parameters	[53]
Scored quantities	Dose to material, proton LET	
Number of histories	1 × 10^9^ initial protons	

**Table 2:** Geant4 simulation parameters.

Parameter	Description	References
Code release date	Geant4.10.6.p01	[55]
Validation	ICRU 73 stopping powers incorporated into Geant4 including media such as water.	[56]
CPU description	High-performance cluster, 40 parallel computations of 1 h on 36 cores	
Source description	Pencil beams modeled as Double Gaussian functions: (1) 75% intensity with a standard deviation 2 mm, (2) 25% intensity with a standard deviation of 4 mm for wide-scattered protons	
Cross-sections	G4HadronElasticPhysics and emstandard_opt4	[56,57]
Transport parameters	Multiple Coulomb scattering based on Lewis theory using the Urban model	[58,59]
Scored quantities	Energy deposition in material	
Number of histories	(a) a single proton pencil beam, and (b) 75 × 75 proton pencil beams evenly spaced 2 mm apart in a square pattern, with 4 × 10^5^ initial protons per pencil beam	

**Table 3:** Properties of glass scintillator samples.

Sample	Density (g cm^−3^)	Base Glass Composition	Activation Element	Peak-Plateau Ratio	Relative Luminosity (% BGO)	Birks Correction Factor (mg MeV^−1^ cm^−2^)
S1	5.6	21% Gd_2_O_3_, 35% WO_3_, 40% 2H_3_BO_3_	4% Tb_2_O_3_	1.5	3%	30
S2	5.6	21% Gd_2_O_3_, 35% WO_3_, 40% 2H_3_BO_3_	4% Eu_2_O_3_	1.6	6%	22
S3	5.9	21% Gd_2_O_3_, 45% WO_3_, 32% 2H_3_BO_3_	2% Tb_2_O_3_	1.6	2%	33
S4	5.8	22% Gd_2_O_3_, 45% WO_3_, 30% 2H_3_BO_3_	3% Eu_2_O_3_	1.6	5%	28
S5	4.3	30% PbO, 66% 2H_3_BO_3_	3% Tb_2_O_3_	1.5	4%	30
S6	4.5	30% PbO, 67% 2H_3_BO_3_	4% Eu_2_O_3_	1.5	8%	21

## Data Availability

The raw data supporting the conclusions of this article will be made available by the authors on request, following an embargo from the date of publication to allow for commercialization of research findings.
